# New Model for Quantifying the Nanoparticle Concentration Using SERS Supported by Multimodal Mass Spectrometry

**DOI:** 10.1021/acs.analchem.2c03779

**Published:** 2023-01-26

**Authors:** Aristea Anna Leventi, Kharmen Billimoria, Dorota Bartczak, Stacey Laing, Heidi Goenaga-Infante, Karen Faulds, Duncan Graham

**Affiliations:** † Department of Pure and Applied Chemistry, Technology and Innovation Centre, University of Strathclyde, 99 George Street, GlasgowG1 1RD, U.K.; ‡ National Measurement Laboratory, LGC, Teddington, MiddlesexTW11 0LY, U.K.

## Abstract

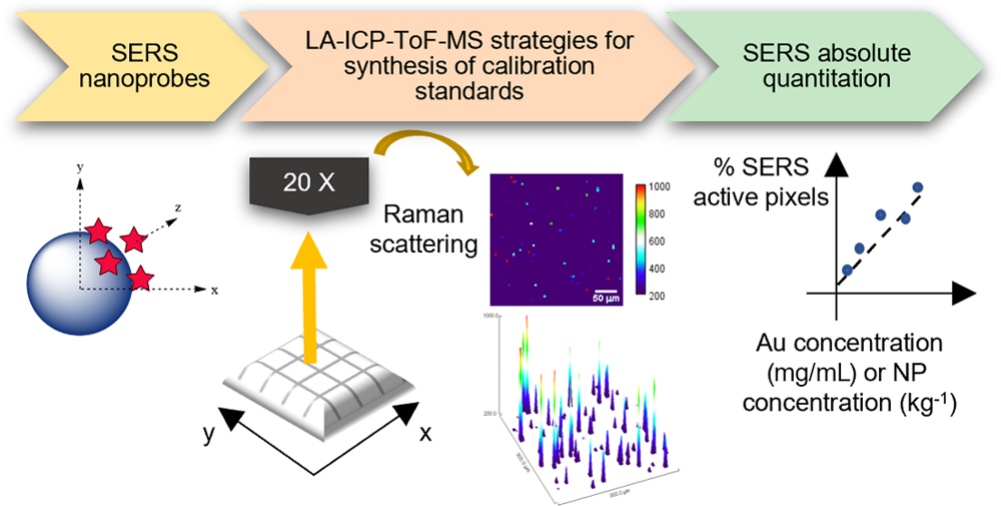

Surface-enhanced Raman scattering (SERS) is widely explored for the elucidation of underlying mechanisms behind biological processes. However, the capability of absolute quantitation of the number of nanoparticles from the SERS response remains a challenge. Here, we show for the first time the development of a new 2D quantitation model to allow calibration of the SERS response against the absolute concentration of SERS nanotags, as characterized by single particle inductively coupled plasma mass spectrometry (spICP-MS). A novel printing approach was adopted to prepare gelatin-based calibration standards containing the SERS nanotags, which consisted of gold nanoparticles and the Raman reporter 1,2-bis(4-pyridyl)ethylene. spICP-MS was used to characterize the Au mass concentration and particle number concentration of the SERS nanotags. Results from laser ablation inductively coupled plasma time-of-flight mass spectrometry imaging at a spatial resolution of 5 μm demonstrated a homogeneous distribution of the nanotags (between-line relative standard deviation < 14%) and a linear response of ^197^Au with increasing nanotag concentration (*R*^2^ = 0.99634) in the printed gelatin standards. The calibration standards were analyzed by SERS mapping, and different data processing approaches were evaluated. The reported calibration model was based on an “active-area” approach, classifying the pixels mapped as “active” or “inactive” and calibrating the SERS response against the total Au concentration and the particle number concentration, as characterized by spICP-MS. This novel calibration model demonstrates the potential for quantitative SERS imaging, with the capability of correlating the nanoparticle concentration to biological responses to further understand the underlying mechanisms of disease models.

## Introduction

Surface-enhanced Raman scattering (SERS) is a non-destructive vibrational spectroscopic technique offering molecularly specific analysis for various applications in the fields of chemical, material, and life sciences.^1−3^ Since its discovery in 1974,^4^ SERS has been in the spotlight of scientific interest as it provides additional enhancement to conventional Raman signals. By incorporating a metallic nanostructure that supports localized surface plasmon resonances, the Raman signal arising from analytes adsorbed onto, or in close proximity to, the metal is increased by several orders of magnitude.^5^ Due to the high sensitivity and selectivity offered, SERS has become a valuable tool in the identification and quantification of analytes in complex matrices such as cells or tissues.^6^

Although significant milestones have been reached within the SERS community, such as single-molecule sensitivity^7−9^ and multiplexing capabilities,^10−12^ the potential of achieving absolute quantitation remains a challenge.^13,14^ The quantitation process can be adversely affected by a variety of factors and therefore can be challenging. SERS has a near-field effect, meaning that only molecules located at a very small distance (a few nanometers) from regions of high electrical fields, known as “hot spots”, are predominantly responsible for the observed SERS signal.^15^ As quoted by Bell et al.^14^ in a review emphasizing the challenges associated with SERS quantitation, “it is important to note that what we are seeing, sensing, targeting and studying in SERS is usually a tiny fraction of the total adsorbed molecules”. Therefore, even with a uniform distribution of analyte molecules around the metallic surface, the number of hot spots can vary depending on the surrounding particles, and this leads to a non-uniform SERS response from each nanoparticle. The variable nature of signal enhancements provided by the nanomaterials is a critical parameter affecting the robustness of quantitation. Currently, the best approach to accommodate the different levels of enhancements provided by the nanoparticles is to interpret the average signal from many molecules and hot spots.^16^ Analyte adsorption properties, such as analyte efficiency and concentration (surface coverage) and binding mode and orientation (fixed or random), can further influence the SERS signal.^15^ Moreover, the experimental setup must be controlled to minimize small changes in conditions such as temperature, laser power, and focus to further reduce the variation in the signal.^14,15^

To overcome some of these challenges, researchers have incorporated the use of internal standards (IS) to correct the signal variability observed. One of the first examples was the quantification of dipicolinic acid for the indication of potential anthrax attacks, where potassium thiocyanate was used as an IS.^17^ Although the use of IS corrects against the signal variations, there can be unequal competition between the reporter and IS for the metallic surface.^13^ Therefore, even for compounds with similar chemical structures, the unequal competition will lead to non-linear ratios of analyte and IS.^18^ Isotopologues can be used as IS to improve this issue as their structure will only differ in the isotopic composition from the analyte, resulting in a better match in signal competition for the metal sites.^13^ The application of isotopologues has been demonstrated for single-molecule detection of crystal violet,^19^ the quantification of markers in human blood serum^20^ and plasma,^21^ as well as the quantification of nicotine in electronic cigarettes.^22^ However, as discussed in a review by Goodacre et al.,^13^ isotopologues might not fully compensate for the competitive co-adsorption onto the metallic nanomaterial between different chemical species, which occurs in complex samples such as clinical samples. In these matrices, selective extraction of the analyte is recommended to reduce interference from other components in the sample matrix.

An alternative approach to improve competitive adsorption issues was reported by Shen et al., where the authors used core-shell nanomaterials.^23^ These structures have a molecular layer that contains the IS, which is protected from the surrounding environment, leading to improved quantitative analysis. However, these core-shell substrates still demonstrate batch-to-batch variability.^18^ The use of the standard addition method (SAM) has also been investigated for quantitative analysis, where adding increasing concentrations of a target molecule allowed a calibration curve between the SERS intensity and concentration to be obtained, which was then used to predict the analyte concentration in unknown samples.^13^ The work of Hidi et al.^24^ demonstrated the combination of SAM with lab-on-a-chip SERS devices and applied it for the quantification of methotrexate. The same group also reported the quantification of the analyte Congo red,^25^ followed by the antibiotic nitroxoline^26^ and the analyte nicotine in the presence of cotinine and anabasine in human urine.^27^

Despite the development of these methods, the lack of a definitive approach illustrates the urgent need for well-characterized standards for SERS quantification, which have the capability to be applied in complex biomatrices such as cells or tissues. While other SERS-based quantitative approaches are focused on calibration of the reporter concentration, we report a novel approach for progressing toward absolute quantification. This work demonstrates the potential of a multimodal platform consisting of a single particle inductively coupled plasma mass spectrometry (spICP-MS)^28^ and fast laser ablation inductively coupled plasma time-of-flight mass spectrometry (LA-ICP-ToF-MS) imaging approach to support the absolute quantitation of SERS signals.

Suitable SERS nanotags were synthesized and characterized using spICP-MS providing the Au mass concentration and particle number concentration, which were used to create the calibration model. Similarly to SERS, LA-ICP-ToF-MS imaging is a well-established bioimaging technique that is commonly applied for the detection of nanomaterials in cells and provides spatially resolved information on the elemental distribution of interest within the sample.^29^ If correct calibration strategies are adopted, LA-ICP-ToF-MS can offer the added benefit of quantitative results.^30^ Due to the lack of certified reference materials available for quantification, matrix-matched standards resembling the biochemical environment of the interrogated sample are commonly prepared by spiking ionic standards into the matrix.^30^ Gelatin has been successfully used in previous work for calibration standard preparation as its composition closely matches that of a typical biological tissue or cell sample.^31^ In this work, conventional methods used in LA-ICP-ToF-MS for the preparation of calibration standards were adopted to meet the requirements of SERS measurements. The synthesis of calibration standards containing the SERS nanotags was demonstrated herein, and a novel bioprinting approach reported by Billimoria et al.^32^ was adopted to implement fast synthesis of gelatin droplets. These droplets were designed to mimic the biochemical environment of typical biological models such as cells or tissues. After ensuring their stability and homogeneity at the 5 μm spatial resolution as monitored by LA-ICP-ToF-MS, the standards were spatially mapped by SERS. Univariate and multivariate analysis methods were explored for extracting quantitative SERS information. The assessment of the SERS response was conducted by an “active-area” approach previously reported by Kapara et al.,^33^ which considers the pixels containing nanotags with respect to the total number of pixels mapped and calculates the percentage of SERS active pixels for the development of a quantitation model. This approach successfully calibrates the SERS response against the total Au concentration obtained by spICP-MS and provides a step toward absolute quantitation.

## Experimental Section

### Materials

1,2-bis(4-pyridyl)ethylene (BPE) and gelatin from porcine skin (gel strength ∼300 g Bloom, Type A) were purchased from Sigma-Aldrich Ltd. (Gillingham, UK). All glassware was decontaminated with aqua regia (3 HCl: 1 HNO_3_) prior to use.

### Synthesis and Characterization of SERS Nanotags

Gold nanoparticles (AuNPs) were synthesized using a citrate reduction method previously reported by Turkevich et al.^34^ Following this, the AuNPs were functionalized with a selected Raman reporter, BPE, to create simple nanotags (BPE-AuNPs). For the functionalization, a target concentration of 100 nM BPE was used as it results in a strong SERS signal without inducing aggregation on the colloidal suspension. The resulting nanotags were extensively characterized by extinction spectroscopy, particle tracking analysis (PTA), and SERS solution analysis. Detailed protocols on their synthesis and characterization can be found in the Supplementary Information.

### Preparation and Characterization of Gelatin Calibration Standards

For the preparation of calibration standards, a 3D printing approach was adopted as reported by Billimoria et al.^32^ Briefly, a 1% (w/w) gelatin solution was prepared and spiked with 25 μL of BPE-AuNPs of increasing concentrations. To ensure a homogeneous composition, the resulting mixtures were thoroughly mixed on a hotplate at 45 °C. The calibration standards were prepared by a CELLINK BiOX6 3D printer (BICO, Göteborg, Sweden) using a pneumatic syringe to deposit gelatin droplets on a chilled glass microscope slide (10 °C) with an extrusion time of 0.03 s and a pressure of 5 kPa at 37 °C. The 3D-printed droplets were dehydrated and produced 2D gelatin sections (1 × 1 mm size). Once prepared, the calibration standards were characterized by LA-ICP-ToF-MS for their stability and homogeneity. Detailed protocols on their characterization can be found in the Supplementary Information.

### Development of the Calibration Model

The calibration model was produced by correlating the obtained SERS signals to the elemental information of the nanotags, as characterized by spICP-MS.

#### spICP-MS Analysis

The BPE-AuNPs were characterized by spICP-MS to obtain the elemental information of the nanotags (Au concentration and particle number concentration) used for the calibration model. An 8900 ICP-QQQ-MS instrument manufactured by Agilent Technologies (California, USA) was used throughout. The instrument was equipped with a micromist nebulizer, a Scott-type double-pass spray chamber cooled to 2 °C, and the MassHunter 4.6 software. An ICP-MS instrument was tuned daily using a 1 μg L^–1^ Agilent tuning solution containing Li, Y, and Tl in order to verify the instrument’s performance. Then, the response factor of the instrument to the element was optimized with 1 μg kg^–1^ of ionic Au in 1 mM trisodium citrate in order to obtain the best sensitivity with a minimum background contribution. Isotope ^197^Au was monitored during measurements. spICP-MS analysis in fast time-resolved analysis mode was performed using a dwell time of 100 μs, with no settling time between the measurements, and using the Single Particle Application Module of the ICP-MS MassHunter 4.6 software. Analysis was performed in a “no gas” mode. The instrument was cleaned with 1 mM trisodium citrate after each sample. The Application Module of the ICP-MS MassHunter 4.6 software, as well as in-house developed Excel spreadsheets, was used for data processing. The particle number concentration (*C*_NP_) in the sample was derived from eq 1, accounting for the sample dilution factor:
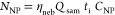
1where *N*_NP_ is the number of nanoparticles detected during the selected acquisition time (*t*_i_), η_neb_ is the transport efficiency, and *Q*_sam_ is the sample uptake mass flow (g min^–1^). The transport efficiency (η_neb_) was determined using the frequency method against the reference material LGCQC5050.^35^ The particle number concentration of the calibration standards in gelatin was calculated taking into account the dilution factor and assuming 99% dehydration.

#### SERS Analysis

A Renishaw InVia Raman confocal microscope equipped with a Leica 20x/NA 0.4 N PLAN EPI objective and a HeNe 633 nm laser excitation source was used. A grating of 1800 l mm^–1^ in high confocality mode and a laser power of 12 mW (100% power) with a 1 s acquisition time per point were used to map gelatin areas. The 2D maps were collected with a spatial resolution of 5 μm in the *X* and *Y* directions. The laser resolution was 1.9 μm as calculated for the selected excitation wavelength and microscope objective.

#### SERS Data Processing

A detailed schematic of the different processing methods can be found in the Supplementary Information section (Experimental Section for SI, ESI, Figure S8). The Windows-based Raman Environment (WiRE – Renishaw plc) 4.4 software was used to pre-process all collected spectra for baseline correction and cosmic ray removal. The 2D SERS maps were imported into the Matlab software. The intensity of the spectral bin at 1610 cm^–1^, corresponding to the key BPE peak, was extracted and exported into Excel. The resulting matrix consisted of 60 rows by 60 columns, corresponding to the 60 spectra collected in the *X* and *Y* directions. This process was repeated for all calibration standards with *n* = 2 replicates per condition. The data sets corresponding to blank samples, one originating from gelatin alone and the other from gelatin spiked with bare AuNPs, were used for understanding the behavior of scattering when no SERS nanotags were involved. From their histograms, the threshold for SERS active pixels was set as the mean value of the blank samples. The percentage of SERS active pixels was calculated per standard, as shown in eq 2, and plotted using OriginPro (2020) against the total Au concentration or particle number concentration, as characterized by spICP-MS. Linear regression was used to fit the data.
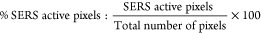
2

## Results and Discussion

The concept proposed is to correlate the SERS response in a 2D Raman map with the Au concentration of nanotags as validated by spICP-MS to provide absolute quantitation from the optical SERS measurements. This was achieved by preparing SERS nanotags that were then quantified in a gelatin matrix by allowing the subsequent SERS intensities from a Raman mapping experiment to be calibrated to absolute concentrations of the SERS nanotags.

### Characterization of SERS Nanotags

AuNPs, a commonly adopted SERS substrate, were selected for this work in order to develop nanotags that can be widely tested and offer a biocompatible and non-toxic nature. AuNPs were synthesized using a citrate reduction method previously reported by Turkevich et al.^34^ and functionalized with a Raman reporter, BPE. The reporter produces a strong “fingerprint” SERS spectrum with a characteristic peak at 1610 cm^–1^ (ESI, Figure S1). The reporter concentration was optimized to produce a strong SERS signal without inducing aggregation and colloidal instability. The selected BPE concentration was 100 nM (ESI, Figure S2). The surface coverage did not reach a complete monolayer of occupied binding sites as this could result in colloidal instability. For reporters bound to the nanoparticle by the pyridyl group such as BPE, the band around 1600 cm^–1^ is enhanced upon adsorption to the metal and is a result of the aromatic ring C–C/N stretching.^36,37^ The nanotags (BPE-AuNPs) were then characterized by extinction spectroscopy and PTA to determine whether they were stable and met the design criteria. The results revealed a monomodal character with minimal presence of aggregates and an average size of 36 ± 2.6 nm (ESI, Figure S3). The colloidal stability and minimal presence of aggregates were also validated by spICP-MS measurements (ESI, Figure S4).

### Relationship between BPE and Au in the Nanotags

In order to develop a calibration model relating the SERS signal intensity to the elemental concentration of the nanoparticles, it was important to further characterize the nanotags and consider the relationship between BPE and Au (ESI, Figure S5). The uniformity of the bulk SERS signal across different batches of nanotags of the same core AuNPs was explored by monitoring the relative standard deviation (RSD) of the BPE peak at 1610 cm^–1^ using 638 nm laser excitation. The investigation verified one of the key challenges in obtaining quantitative SERS results, batch-to-batch variability. The source of signal variation originates from the intrinsic nature of SERS, where multiple parameters can affect the absolute SERS intensity. Experimental factors such as instrument performance and laser fluctuations can be accounted for by standardizing the signal obtained and reporting a relative SERS value. Even with this instrumental calibration, the reported RSD between batches (31.4%, *n* = 7) suggests poor reproducibility. This is a common observation in the field of SERS and potentially originates from the uncontrolled presence of hot spots and the various enhancements produced from the nanoparticles in the colloidal suspension. That being said, the irreproducibility could potentially be reduced by introducing large batch synthesis rather than multiple small batches as well as introducing more control over the addition of BPE to the nanoparticles. Another factor influencing the presence of hot spots can be the method of adding the reporter. Small fluctuations in the way the BPE stock is introduced to the colloidal suspension can potentially cause a higher number of hot spots around the particles that first interact with the reporter. However, it should be noted that within the same batch of BPE-AuNPs, the RSD values are extremely low (<1%, *n* = 3), meaning that within the same batch of nanoparticles, the signal obtained is repeatable.

In contrast, analysis by spICP-MS showed a uniform Au signal across all batches analyzed. The particle mass concentration, as characterized by spICP-MS, also followed the same trend across all batches (ESI, Figure S6). This supports the hypothesis that the variation in the BPE signal is not due to the AuNP synthesis process but rather a result of the interactions between BPE and the nanoparticles, which are challenging to control. To overcome this challenge, the characterization steps and collected measurements were made using a single batch of nanotags (ESI, Figure S5, BPE-AuNPs batch 7, BPE/Au 0.83:56.2).

### Synthesis and Characterization of Gelatin-Printed Calibration Standards

To produce a suitable calibration standard, a matrix that mimics the biochemical composition of a biological environment was necessary. The preferred matrix for this purpose was gelatin as it is well established and its composition is representative of a typical biological tissue or cell sample.^31^ Briefly, a 1% (w/w) gelatin solution was prepared and spiked with increasing concentrations of BPE-AuNPs. A 3D printing approach reported by Billimoria et al. was implemented for fast and controlled synthesis of gelatin droplets (1 × 1 mm in size).^32^ The resulting droplets were dehydrated, producing thin gelatin sections suitable for 2D SERS analysis.

**Figure 1:**
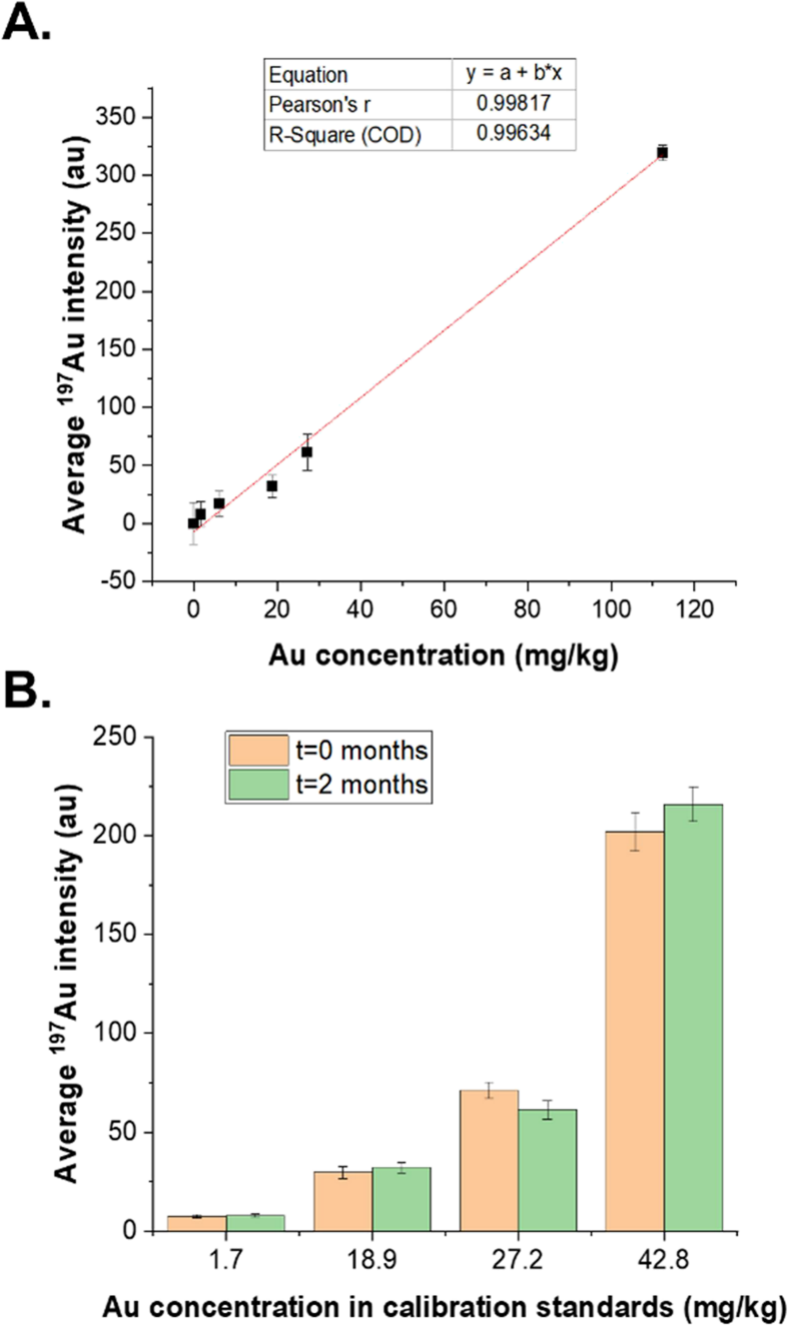
Characterization of calibration standards by LA-ICP-ToF-MS. A. Linear increase of the ^197^Au intensity with the Au concentration. Error bars represent ± RSD of intensity (*n* = 3 replicates). B. Stability of calibration standards after two months of synthesis. Error bars represent means of two (*t* = 0 months) or three (*t* = 2 months) replicates ± average between-line SD.

Similarly to SERS, LA-ICP-ToF-MS imaging is a well-established bioimaging technique that is commonly applied for the detection of nanomaterials in cells and provides spatially resolved information on the elemental distribution of interest within the sample.^29^ Therefore, LA-ICP-ToF-MS imaging was used to support the development of suitable calibration standards. The analysis of these standards with a 5 μm spatial resolution demonstrated the homogeneous distribution of nanotags across the gelatin droplets (ESI, Figure S7; between-line RSD < 14%). Moreover, the ^197^Au response was found to increase linearly with increasing Au concentration in the standards (*R*^2^ = 0.99634) (Figure 1A). Furthermore, the stability of calibration standards was confirmed, and the ^197^Au intensity remained constant after two months of synthesis (Figure 1B). Overall, the characterization by LA-ICP-ToF-MS confirmed the homogeneity and stability of the calibration standards and validated their suitability for quantitation purposes.

### Development of the SERS Calibration Model

The same gelatin calibration standards were analyzed by SERS in order to develop a calibration model. Spatial Raman mapping was performed with a 633 nm laser for large areas (300 μm × 300 μm) of the gelatin droplets at a 5 μm resolution with a 1 s integration time per point. The sample size ensured that a representative area per condition was imaged with 7200 spectra evaluated per calibration standard. Each laser position in the sample map produced a SERS spectrum, creating a 2D data set, where one spectrum correlates to one pixel in the resulting image.

**Figure 2:**
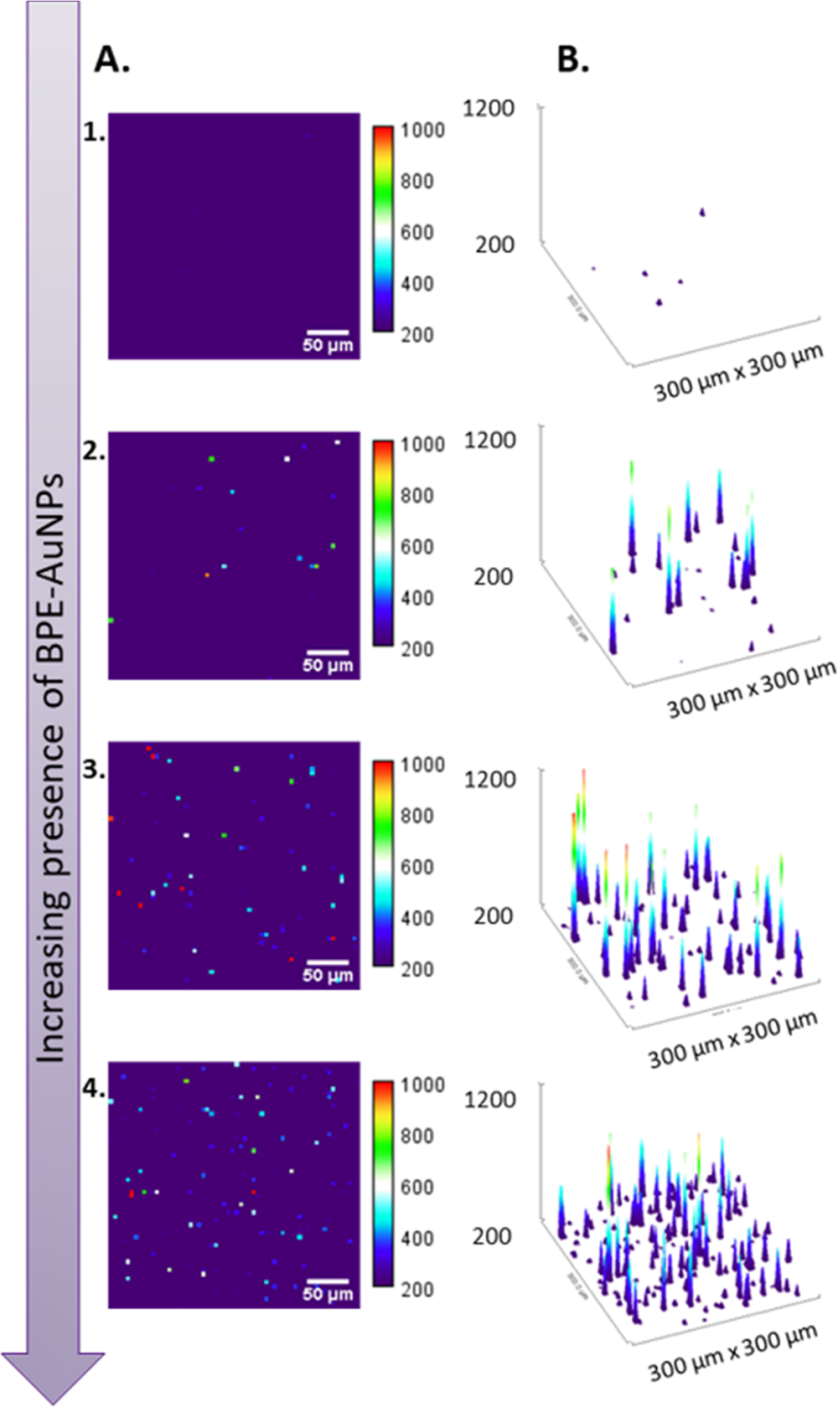
Assessment of the SERS response by an “active-area” approach, where the number of SERS active pixels increases with the increased presence of BPE-AuNPs in the calibration standards. A. False color images representing the absolute SERS intensity of the BPE peak at 1610 cm^–1^. Samples 1–4 are of increasing BPE-AuNPs presence (1: 0 kg^–1^, 2: 1 × 1013 kg^–1^, 3: 7.0 × 1013 kg^–1^, and 4: 1.8 × 1014 kg^–1^). The color balance was adjusted, selecting a minimum value of 200 to account for the matrix contribution and removing pixels related to the background. B. Surface plots illustrating the number of SERS events, corresponding to the false color images depicted in column A. The height of the spikes is relevant to the color scale shown in column A, while the number of spikes increases with increasing amounts of BPE-AuNPs and therefore “active pixels”. One pixel corresponds to one spectrum collected. Sample 1 contains a few pixels with absolute values close to 200, which is the reason why small spikes are shown on the surface plot.

Understanding the nature of the SERS signal in 2D mapping experiments is critical to developing a fit-for-purpose calibration model. The signal originates from the reporter molecule adsorbed on the metallic nanostructure that experiences a surface enhancement producing a SERS signal, where its intensity is dependent on the reporter concentration. Since the calibration standards produced are prepared using the same nanotag batch, they have the same BPE concentration present on the nanotags, and therefore, the absolute SERS intensity of pixels containing BPE-AuNPs should remain constant throughout the calibration standards. However, the total number of nanotags present will increase with higher concentration standards. For this reason, the SERS response was evaluated according to the number of SERS events rather than their absolute intensity. This is an “active-area” approach that considers the pixels containing nanotags with respect to the total number of pixels mapped and calculates the percentage of SERS active pixels for the development of a quantitation model. Therefore, increasing the presence of BPE-AuNPs in gelatin will result in a higher number of events (Figure 2). Fluctuations in the intensity are expected as they depend on various enhancements produced by the nanoparticles, the presence of hot spots, as well as the uniformity of the distribution of nanotags in the supporting matrix. Moreover, the laser spot diameter is a limiting factor because it is larger than the nanoparticles, and therefore, the collected spectrum can be a result of multiple nanoparticles captured under one laser pulse, again making quantitation challenging. It is also important to note that the 3D-printed gelatin droplets are dehydrated, leading to 2D-like structures, where the thickness of the droplet is assumed constant. Thickness fluctuations, although not expected, could influence the sample focus and affect the resulting SERS signal.

The collected maps were evaluated by two processing methods, based on either univariate or multivariate analysis. A schematic illustration of the data analysis methods can be found in the Supplementary Information (ESI, Figure S8). The key difference between the two models is the way the system recognizes and classifies a pixel as SERS active. Univariate analysis (method A) bases this selection on the Raman peak of BPE at 1610 cm^–1^, whereas multivariate analysis (method B) uses the whole spectrum collected and identifies the level of overlap with a BPE reference spectrum. The latter offers a greater level of confidence in identifying active pixels as more spectral features are required for a positive match. However, small variations from the reference spectrum can result in a poorer degree of overlap and therefore potential false negative pixels. In addition, this approach supports relative SERS intensities as the values reported correspond to a color intensity gradient. Therefore, the SERS response cannot be assessed in an absolute manner. On the contrary, method A provides a data set with an absolute value for each pixel that corresponds to the Raman intensity of the 1610 cm^–1^ spectral bin. For this reason, we report method A that allows the investigator to understand the behavior of the matrix and other components as each pixel mapped is associated with an absolute value. Statistical analysis and, more specifically, histograms can represent the behavior of nanotags in different conditions. Control samples consisting of gelatin or gelatin spiked with bare AuNPs were used to understand the contribution of the matrix and scattering features originating from other components (ESI, Figure S9). A Gaussian distribution of pixel intensities (5% skew) with a mean value of 43 counts was determined to be the contribution of control samples. This contribution was consistent in all control samples (RSD < 2%, *n* = 4) and can potentially originate from the data processing steps (ESI, Figure S8, steps 2 and 3). For example, when extracting the intensity values to create a 2D data set equivalent to the presence of BPE-AuNPs (step 3), the value reported corresponds to a small spectral bin instead of the maximum of the 1610 cm^–1^ peak, which can potentially change by a few cm^–1^ between collected spectra. Therefore, the absolute intensities in the matrix can potentially have a negative value. However, the matrix contribution did not interfere with the quantitation process as it was taken into account when determining the threshold for SERS “active” pixels.

**Figure 3:**
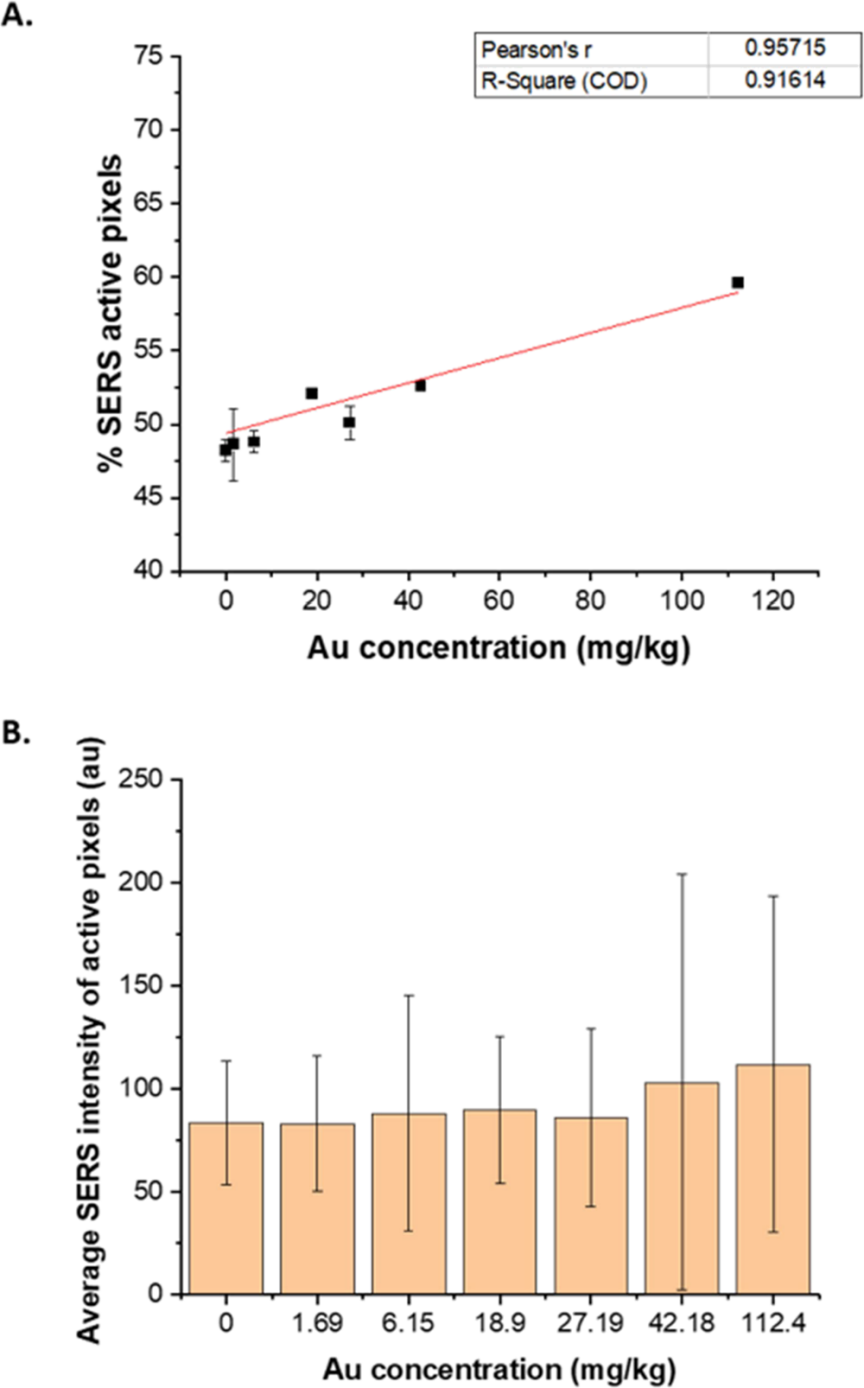
A. Reported calibration curve showing a linear increase between the percentage of SERS active area and the Au concentration present in the standards. Error bars correspond to mean ± SD (*n* = 2 replicates per condition with 3600 spectra per replicate). B. On the contrary, the absolute intensity of SERS active pixels does not increase with the Au concentration. Instead, it depends on the concentration of the Raman reporter present in the nanotags and can fluctuate due to the presence of “hot spots” and the various enhancements produced by the nanoparticles. Error bars represent ± standard deviation.

When investigating the percentage of SERS active pixels with respect to the Au concentration, a greater level of linearity is achieved when the threshold is set as the mean value of the blank samples (ESI, Figure S8, method A.1.1) compared to the maximum value of blank samples (ESI, Figure S8, method A.1.2). This observation underlines the importance of accepting the intrinsic nature of the SERS signal obtained. It is a complex collection of scattering events originating from multiple components, and by selecting a higher threshold to exclude active pixels from the blank samples, spectral information not visible to the naked eye can be lost. For these reasons, we have reported method A.1.1 (Figure 3A) that calibrates the SERS response against the Au concentration. The *x* axis can be alternatively converted to the particle number concentration, as characterized by spICP-MS (ESI, Figure S10). Other methods investigated support each other with predictions that are within the experimental error of ±25% (ESI, Figure S11). This novel calibration model correlates the SERS response to the total Au concentration, as characterized by spICP-MS. Quantification with respect to the nanoparticle concentration instead of the Raman reporter can provide a more “absolute” approach, where the measurements are traceable and comparable to other analytical techniques. This absolute quantitative capability can potentially allow the correlation of the nanoparticle concentration to biological responses to further understand the underlying mechanisms of the interaction of nanotags with disease models.

## Conclusions

A quantification model for SERS has been developed by correlating the obtained SERS signals to the elemental information of the nanotags, as characterized by spICP-MS. The SERS nanotags were introduced in gelatin, and calibration standards were created using a bioprinting approach. By using LA-ICP-ToF-MS, the standards were found to be sufficiently homogeneous (between-line RSD < 14%) and stable for quantitative purposes. The surface of the standards was spatially mapped by SERS with a 633 nm laser beam at a resolution of 5 μm, with a total of 7200 spectra collected per standard. The collected maps were analyzed by classifying the respective pixels according to SERS positive or negative events. The response was then calibrated against the concentration of AuNPs present in gelatin (as determined by spICP-MS and assuming 99% weight loss via dehydration); a property particularly useful as the Raman reporter that provides the detected SERS signal covers only a small percentage of the nanoparticle surface. Various processing methods for assessing the SERS response were investigated, including multivariate and univariate analysis. The proposed method provides an absolute approach for evaluating the SERS response, where each pixel value corresponds to the intensity of the 1610 cm^–1^ spectral bin. The developed quantitation model correlated the SERS response to the concentration of Au originating from the BPE-AuNPs (*R*^2^ = 0.91614).

This work provides an important advance in the development of absolute quantitation models in SERS, where the measurements obtained are correlated to the concentration of nanoparticles. In addition, this work discusses the intrinsic nature of SERS signals and how their unique selectivity and sensitivity can be used for getting closer to achieving absolute quantitation. The proposed model offers a first step toward achieving this and has the potential to be further explored and improved by employing computational methods to identify more parameters for assessing the SERS response and to produce faster and more accurate quantitative outputs. Due to the biologically relevant nature of the gelatin standards, the proposed model has the potential to be combined with bioimaging applications for the absolute quantification of nanoparticles in 3D biological models such as cells or tissues. A potential outcome of this coupling can be the quantification of the targeting effect of nanoparticles on cells or tumors based on the SERS response.

## Data Availability

Research data associated with this work will become available through the following link: https://doi.org/10.15129/3ab2321a-1ca5-486d-8571-2a7683b39a6f.

## Supporting Information Available

Methods for the synthesis of AuNPs and BPE-AuNPs; characterization of BPE-AuNPs by extinction spectroscopy, PTA, and solution SERS; methods for the synthesis of calibration standards; characterization of calibration standards by LA-ICP-ToF-MS; schematic illustration of BPE-AuNP synthesis; optimization of the BPE reporter concentration; characterization of BPE-AuNPs; analysis of BPE-AuNPs by spICP-MS showing a monomodal character; batch-to-batch variation of the SERS signal and total gold mass fraction in the BPE-AuNPs; particle number concentration as characterized by spICP-MS; homogeneity of the ^197^Au intensity across the standards indicating the uniform distribution of nanotags; schematic illustration of the investigated methods for processing the obtained SERS data and developing a calibration model; calibration of SERS signals against the particle number-based concentration; and comparison of the predicted Au concentrations per calibration method (PDF)
