# Supplementary material for: New Model for Quantifying the Nanoparticle Concentration Using SERS Supported by Multimodal Mass Spectrometry
Source: Anal Chem. 2023 Jan 26;95(5):2757–64. doi: 10.1021/acs.analchem.2c03779 (PMC9909670; doi:10.1021/acs.analchem.2c03779)
Supplement: Supplementary file 1 — ac2c03779_si_001.pdf [file ac2c03779_si_001.pdf]

Supporting Information

For

A new model for quantifying nanoparticle concentration using SERS supported by multi-modal mass spectrometry

Aristea Anna Leventi, [1,2] Kharmen Billimoria, [2] Dorota Bartczak, [2] Stacey Laing, [1] Heidi Goenaga-Infante, [2] Karen Faulds [1] and Duncan Graham [1]*

[1] Department of Pure and Applied Chemistry, Technology and Innovation Centre, University of Strathclyde, 99 George Street, Glasgow, G1 1RD, UK.
[2] National Measurement Laboratory, LGC, Teddington, Middlesex, TW11 0LY, UK.
**Contact email: duncan.graham@strath.ac.uk*

Table of contents

Experimental section for SI S2
  Synthesis of SERS nanotags S2
  Characterisation of SERS nanotags S2
  Synthesis of gelatin calibration standards S3
  Characterisation of gelatin calibration standards S3
Supporting Figures S4

Experimental section for SI

Synthesis of SERS nanotags

Gold nanoparticles (AuNPs) were synthesized using a citrate reduction method previously reported by Turkevich *et al.*.[37] More specifically, sodium tetrachloroaurate dihydrate solution (10 mL, 15 mM) was dissolved in 490 mL of double distilled dH_2O and heated until boiling under continuous stirring. Sodium citrate tribasic dihydrate (7.5 mL, 26 mM) was then added and the mixture was boiled under continuous stirring for approximately 1 hour. The resulting colloidal solution was then left to cool, maintaining stirring throughout. Following this, the AuNPs were functionalised with a selected Raman reporter, 1,2-bis(4-pyridyl) ethylene (BPE), to create simple nanotags (BPE-AuNPs). For the functionalization, a target concentration of 100 nM BPE was used, as it results in a strong SERS signal without inducing aggregation on the colloidal suspension. Briefly, 100 µL of 1 µM 1,2-bis(4-pyridyl) ethylene (BPE) was added to 900 µL of Au NPs, incubated on a shaker plate for 30 minutes and centrifuged at 6000 rpm for 20 minutes. The resulting pellet was redissolved in 1 mL double distilled dH_2O.

Characterisation of SERS nanotags

Extinction Spectroscopy: Extinction spectra were measured using an Agilent Cary 60 UV-Visible spectrophotometer with Win UV scan V.2.00 software. Initially the system was left to equilibrate at RT, followed by the insertion of poly(methyl methacrylate) (PMMA) disposable plastic micro cuvettes with 500 µL of sample in order to scan wavelengths from 300 – 800 nm. A baseline was obtained using double distilled dH_2O in place of the sample.

Particle Tracking Analysis (PTA): PTA measurements were performed with an NS300 instrument, manufactured by Malvern Panalytical and equipped with a 405 nm diode laser source, sCMOS camera, syringe pump and NTA3.4 software. The PTA instrument was switched on at least 30 minutes before the measurements. All measurements were performed at room temperature. Videos were recorded with a camera level set to 16, over 60 seconds' duration with 10 seconds equilibration time prior to each measurement. Two independent preparations of each sample were measured five times under repeatability conditions (n=20). Measurements were performed in a flow mode, with a syringe pump set to injection speed of 40. The software assumed viscosity of water for the samples. For the analysis of the recorded videos, detection threshold was set to 5. The obtained data were further processed with Excel using modal diameter values generated by the software.

Surface enhanced Raman scattering (SERS) solution analysis: For SERS solution analysis, a Snowy Range CBEx 2.0 handheld Raman spectrometer (Snowy Range Instruments, Laramie WY USA) equipped with a 638 nm laser excitation with a maximum laser power of 40 mV was utilised.

For each measurement, 500 µL of sample was inserted into a glass vial and the SERS spectra were collected using 100% laser power and 1 s accumulation time. For the control measurement, 500 µL of ethanol was inserted into a glass vial and the SERS spectra were collected using the same method. Peak 1.1.112. software was used to acquire the spectra (n=3 replicates within each condition), which were then baseline corrected in Matlab 2014b. The intensity values of the 1610 cm^{-1} peak were extracted in Excel for each sample and the relative SERS intensities were calculated with respect to the ethanol peak at 883 cm^{-1}.

$$N_{NP} = \eta_{neb}\, Q_{sam}\, t_i\, C_{NP}$$

Synthesis of gelatin calibration standards

For the synthesis of calibration standards, a 3D printing approach was adopted as reported by Billimoria *et al.*[32] Briefly, a 1% (w/w) gelatin solution was prepared and spiked with 25 uL of BPE-AuNPs of increasing concentrations. To ensure a homogeneous composition, the resulting mixtures were thoroughly mixed on a hotplate at 45 ºC. The calibration standards were prepared by a CELLINK BiOX6 3D printer (BICO, Göteborg, Sweden), using a pneumatic syringe to deposit gelatin droplets on a chilled glass microscope slide (10 ºC) with an extrusion time of 0.03 s and pressure of 5 kPa at 37 ºC. The 3D-printed droplets were dehydrated and produced 2D gelatin sections (1 x 1 mm size).

Characterisation of gelatin calibration standards

LA-ICP-ToF-MS Analysis: For this study, an ImageBio 266 nm laser ablation system (ESL, Montana, USA) equipped with a TwoVolume3 ablation cell and dual concentric injector was used. The laser ablation system was coupled to an inductively coupled plasma mass spectrometer with a Time-of-Flight mass analyzer ICP-ToF-MS (2R) system (TOFWERK, Thun, Switzerland). The isotope ^{197}Au was measured in no gas mode. For each standard, multiple parallel line scans (30 lines per replicate) were ablated to create 2D distributions of Au, with a 4 J cm^{-2} fluence, 100 Hz repetition rate and 5 µm laser spot size (n=3 replicates per standard). The data were exported to MS Excel where average signal intensities and variations across the data sets were calculated. Furthermore, the average ion-responses of each standard were plotted against the calibration concentrations in Origin Pro (2020) and linear regression tool was used to create a calibration graph.

Supporting Figures

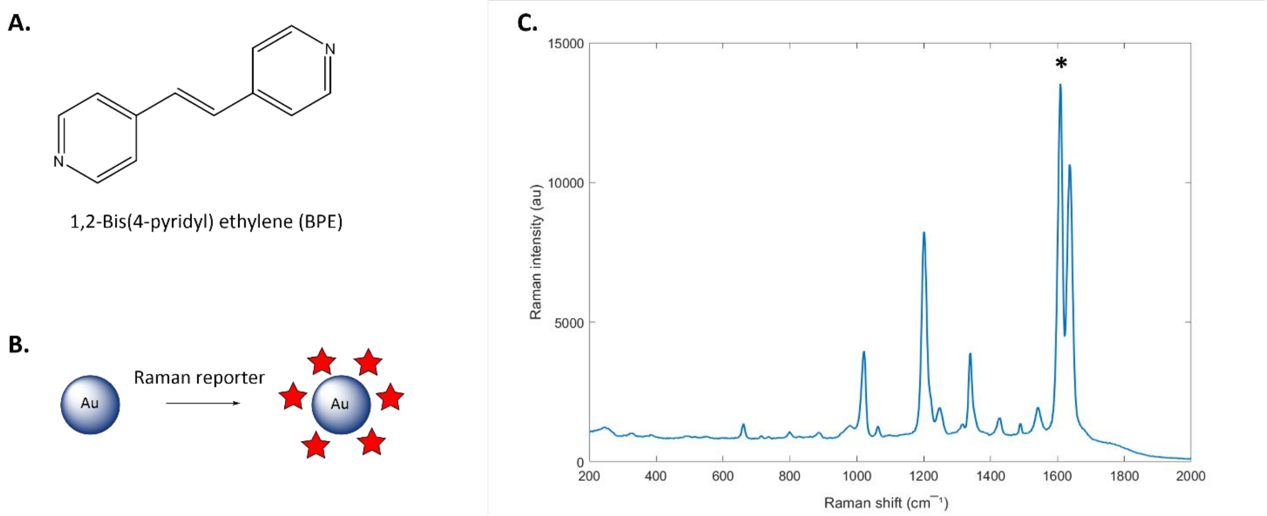

Figure S1. Synthesis of BPE-AuNPs. A. Chemical structure of 1,2-bis(4-pyridyl) ethylene (BPE); the selected Raman reporter. B. Schematic illustration of functionalisation, where BPE is attached to spherical AuNPs by electrostatic interactions. C. SERS spectrum of BPE with a 'fingerprint' peak at 1610 cm^{-1}. A Snowy Range CBEx 2.0 handheld Raman spectrometer (Snowy Range Instruments, Laramie WY USA) equipped with a 638 nm laser excitation with a maximum laser power of 40 mV was used to acquire the spectra (n=3), which were then processed for baseline correction in Matlab. The spectrum shown is the average of the three spectra collected.

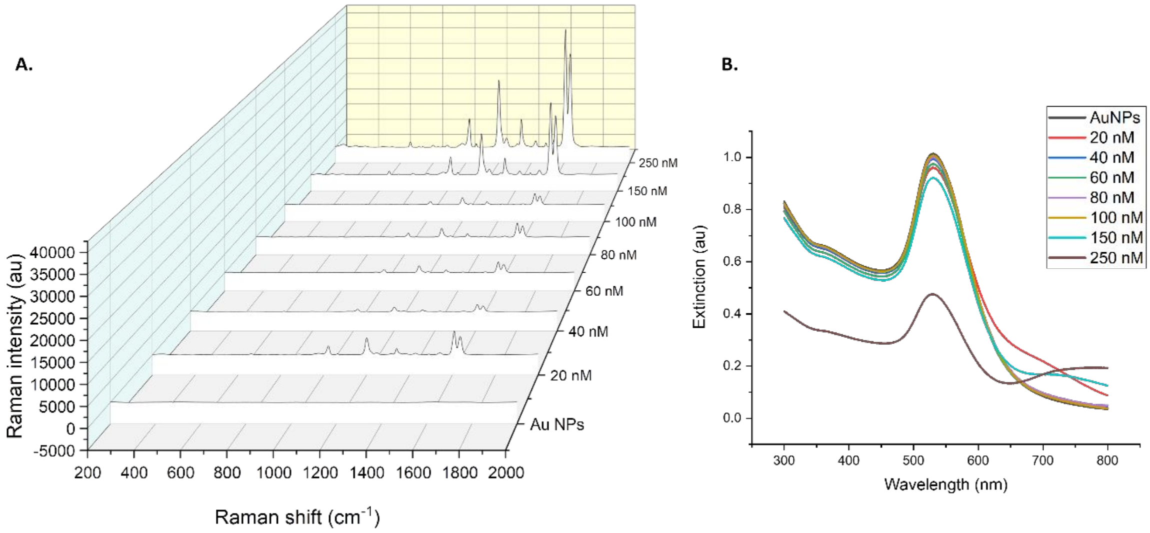

Figure S2. Optimisation of the BPE reporter concentration (0-250 nM) used for the synthesis of BPE-AuNPs. A. SERS spectra collected showing increasing intensity of the 1610 cm^{-1} peak corresponding to BPE. A Snowy Range CBEx 2.0 handheld Raman spectrometer (Snowy Range Instruments, Laramie WY USA) equipped with a 638 nm laser excitation with a maximum laser power of 40 mV and 1 s accumulation time was used to acquire the spectra (n=3), which were then processed for baseline correction and cosmic rays removal in Matlab. The spectrum shown is the average of the three spectra collected. B. Extinction spectra showing the colloidal stability of 20-100 nM BPE-AuNPs and the aggregation present in higher concentrations (150-250 nM).

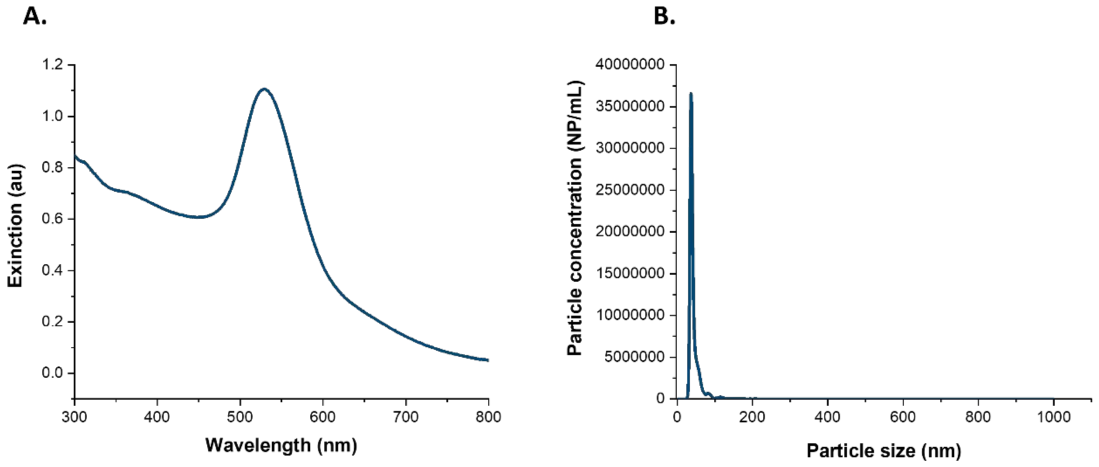

C.

Parameter	Value
λmax (nm)	529
Modal diameter (nm)	36.7± 2.6

Figure S3. Characterisation of BPE-AuNPs. A. Extinction spectra showing the colloidal stability of BPE-AuNPs with an extinction maximum of 529 nm. B. Average PTA size distribution graph, showing a monomodal character with minimal aggregates present (n=20 replicates). C. Summary table with key parameters of the BPE-AuNPs, including λmax and average size. Details on the experimental protocols used to acquire the data can be found in the experimental section.

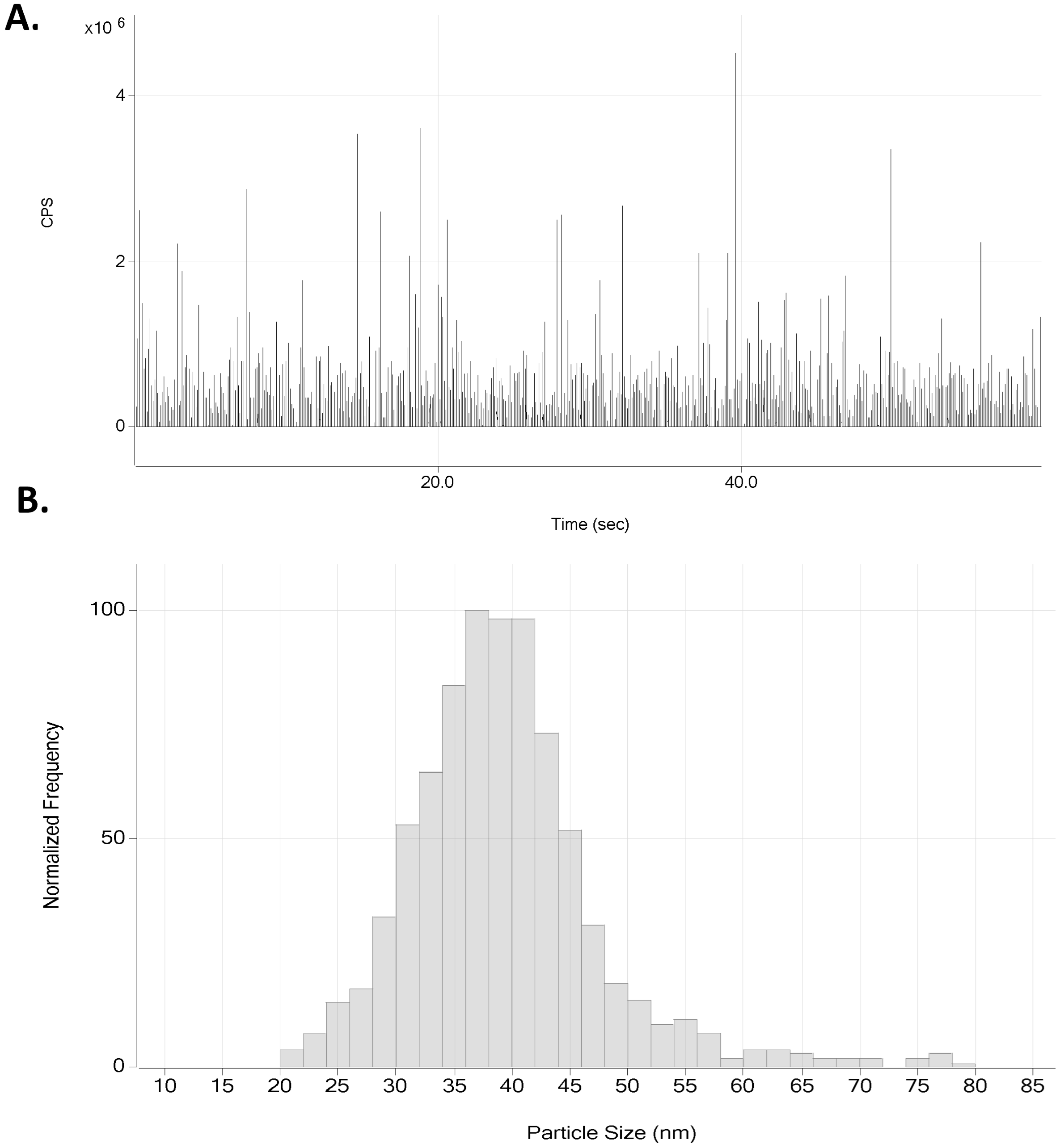

Figure S4. Analysis of BPE-AuNPs by spICP-MS showing a monomodal character with a few aggregates/agglomerates present. A. Representative spICP-MS time scan B. Particle size distribution histogram of the BPE-AuNPs.

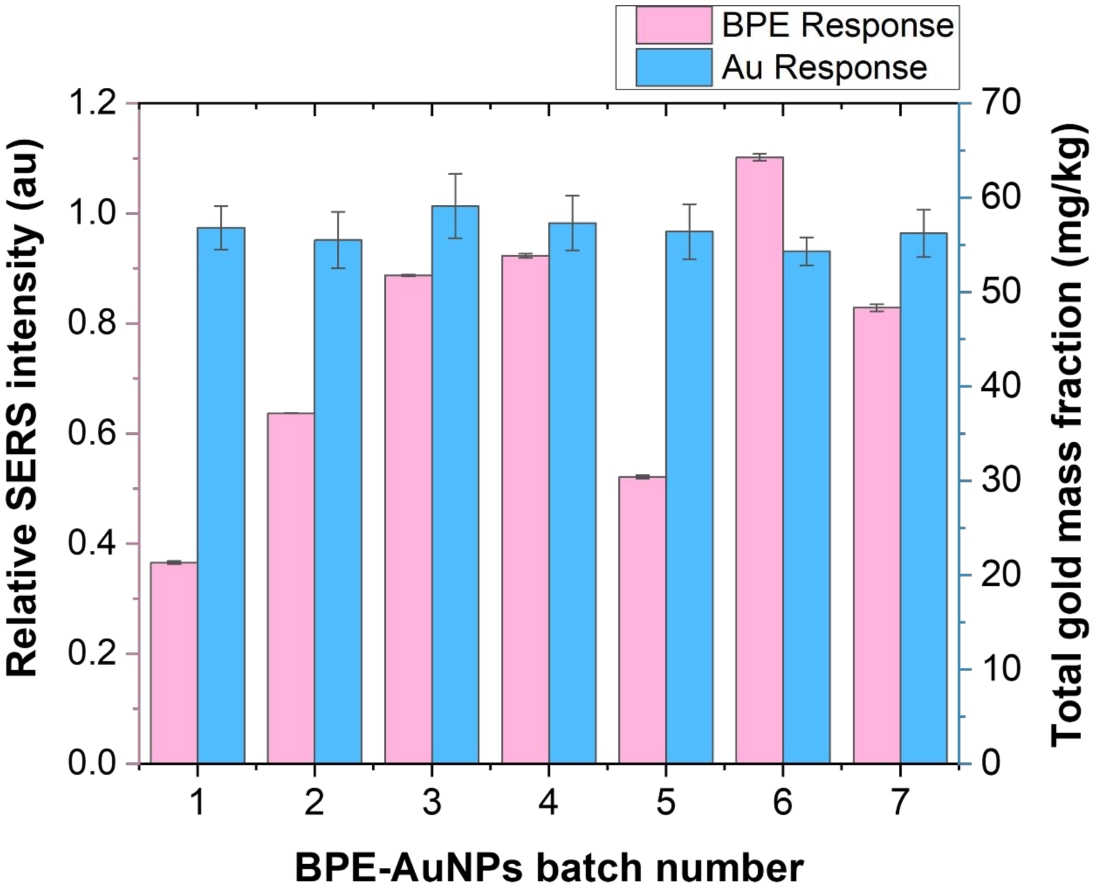

Figure S5. Batch-to-batch variation (n=7 batches) of SERS signal and total gold mass fraction in the BPE-AuNPs. The relative SERS intensity (BPE response, pink) where error bars represent ± SD of the relative SERS intensity at 1610 cm^{-1} (n=3 replicates per batch). The total Au mass fraction (Au response, blue) was measured by spICP-MS and error bars represent ± SD of the total gold mass fraction (n=6 replicates per batch).

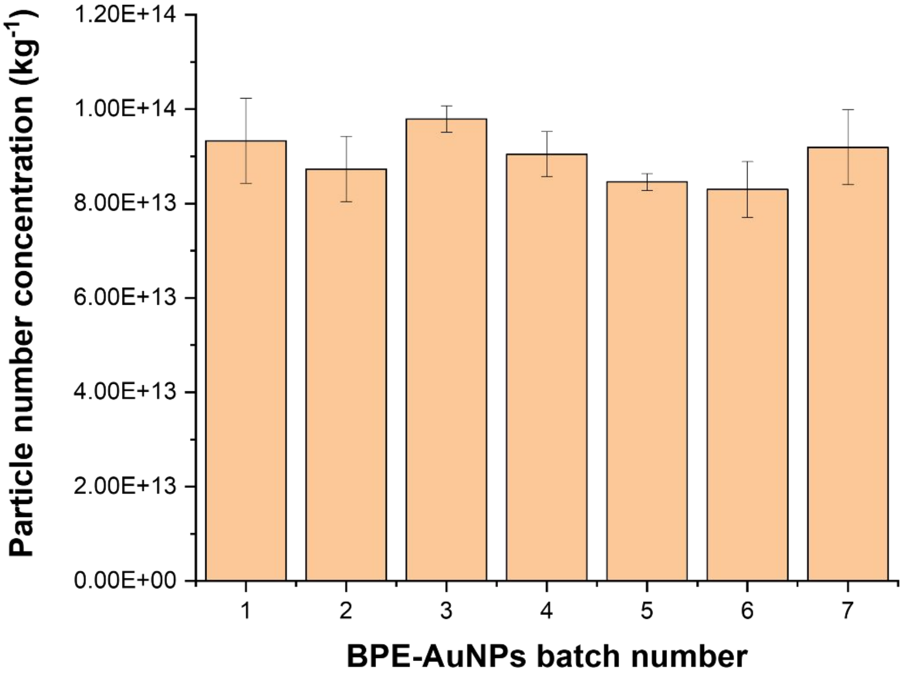

Figure S6. The particle number concentration, as characterised by spICP-MS, remained constant throughout the batches analysed. Error bars represent ± SD of the particle number concentration (n=6 replicates per batch).

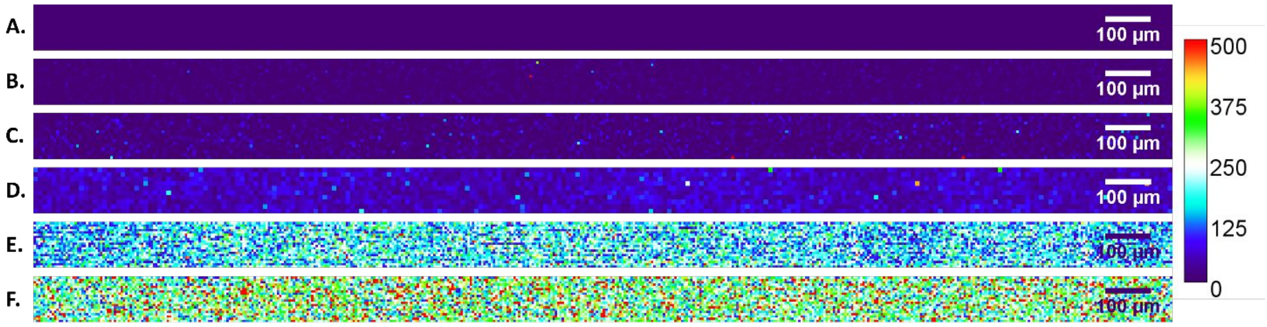

Figure S7. Homogeneity of ^{197}Au intensity across the standards (5 µm spatial resolution), indicating the uniform distribution of nanotags across the gelatin surface (between-line RSD<14%). False colour images were generated using ImageJ with the thermal option on the look up table. The colour balance was adjusted in order to generate a calibration bar applicable for all investigated samples, where A is blank (^{197}Au: 0 mg/kg) and B-F contain increasing amounts of BPE-AuNPs (^{197}Au: 1.69 – 112.40 mg/kg).

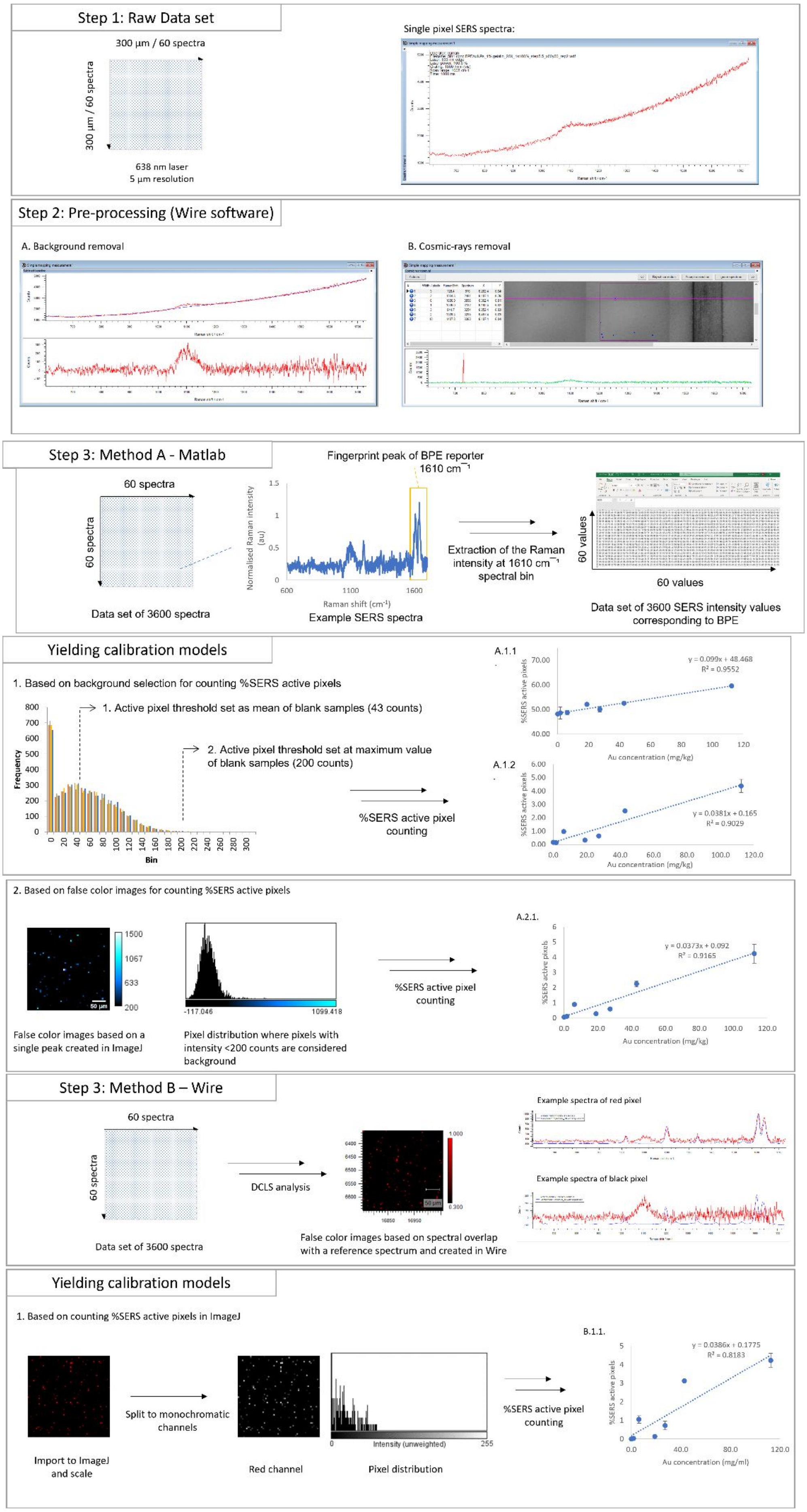

Step 1: Raw Data set
300 µm / 60 spectra
300 µm / 60 spectra
638 nm laser
5 µm resolution
Single pixel SERS spectra:
Step 2: Pre-processing (Wire software)
A. Background removal
B. Cosmic-rays removal
Step 3: Method A - Matlab
60 spectra
60 spectra
Data set of 3600 spectra
Fingerprint peak of BPE reporter
1610 cm⁻¹
Normalised Raman intensity (au)
Raman shift (cm⁻¹)
Example SERS spectra
Extraction of the Raman intensity at 1610 cm⁻¹ spectral bin
60 values
60 values
Data set of 3600 SERS intensity values corresponding to BPE
Yielding calibration models
1. Based on background selection for counting %SERS active pixels
1. Active pixel threshold set as mean of blank samples (43 counts)
2. Active pixel threshold set at maximum value of blank samples (200 counts)
Frequency
Bin
%SERS active pixel counting
A.1.1
y = 0.099x + 48.468
R² = 0.9552
%SERS active pixels
Au concentration (mg/kg)
A.1.2
y = 0.0381x + 0.165
R² = 0.9029
%SERS active pixels
Au concentration (mg/kg)
2. Based on false color images for counting %SERS active pixels
False color images based on a single peak created in ImageJ
Pixel distribution where pixels with intensity <200 counts are considered background
%SERS active pixel counting
A.2.1.
y = 0.0373x + 0.092
R² = 0.9165
%SERS active pixels
Au concentration (mg/kg)
Step 3: Method B – Wire
60 spectra
60 spectra
Data set of 3600 spectra
DCLS analysis
False color images based on spectral overlap with a reference spectrum and created in Wire
Example spectra of red pixel
Example spectra of black pixel
Yielding calibration models
1. Based on counting %SERS active pixels in ImageJ
Import to ImageJ and scale
Split to monochromatic channels
Red channel
Intensity (unweighted)
Pixel distribution
%SERS active pixel counting
B.1.1.
y = 0.0386x + 0.1775
R² = 0.8183
%SERS active pixels
Au concentration (mg/ml)

Figure S8. Schematic illustration of the investigated methods for processing the obtained SERS data and developing a calibration model. Step 1: Understanding the nature of collected spectra. SERS analysis was performed with a 638 nm excitation wavelength and at 5 μm resolution in the X and Y directions, with a total number of 3600 collected SERS spectra per replicate (n=2 replicates per standard). Step 2: Basic pre-processing steps performed in Wire software, including baseline correction and removal of cosmic rays. Step 3: Method A for developing calibration model, which is based on the absolute SERS intensity of the collected spectra at the spectral bin corresponding to 1610 cm^{-1}. The spectra were imported on Matlab, where a script extracted the SERS intensity at the spectral bin corresponding to 1610 cm^{-1}; the area where the BPE 'fingerprint' peak is expected. The resulting 2D data set consisted of 3600 values corresponding to 3600 spectra collected and was extracted to excel for further processing. According to the selection of the threshold for SERS active pixels, calibration models A.1.1 and A.1.2 were generated. Alternatively, false color images were generated in ImageJ, yielding calibration model A.2.1. Step 3: As an alternative, method B is using the whole spectrum for establishing SERS active pixels instead of only the 1610 cm^{-1} region. The collected spectra are imported on Wire after the pre-processing steps and DCLS analysis created false color images based on the overlap of the collected spectra to a reference BPE spectrum. These images were imported to ImageJ and the %SERS active pixels were calculated yielding calibration model B.1.1.

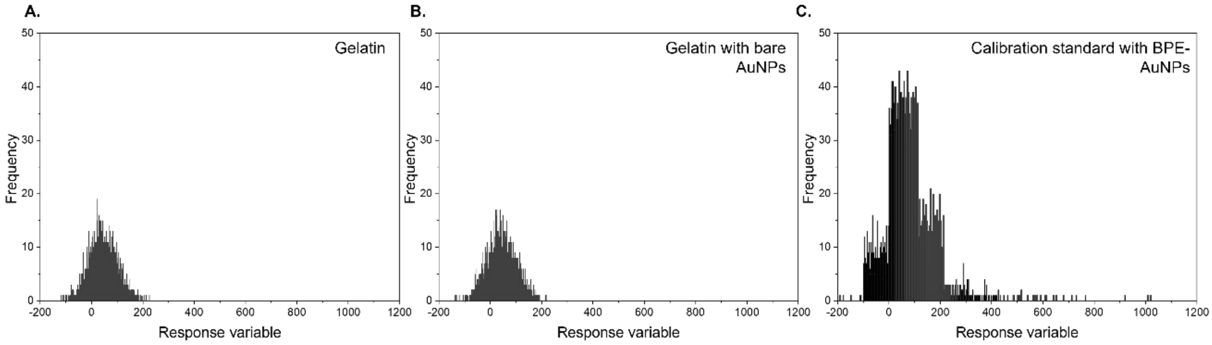

Figure S9. Histograms showing the frequency distribution of pixels in SERS map of gelatin (A) and gelatin spiked with bare AuNPs (B) or the BPE-AuNPs (C).

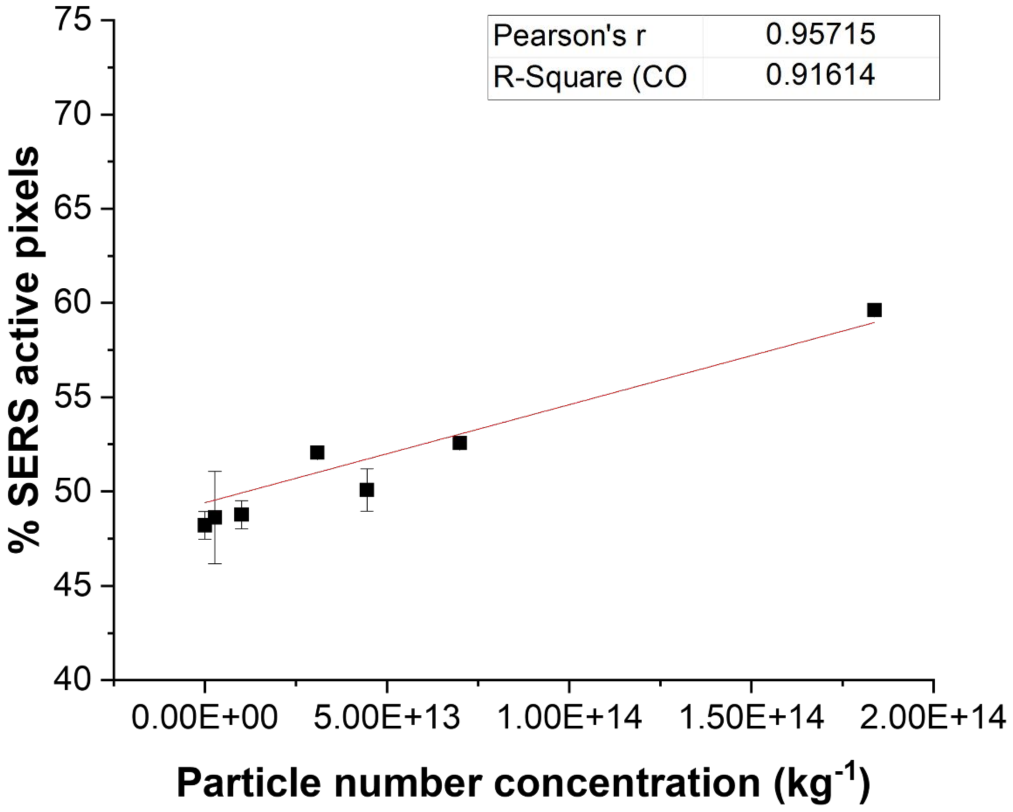

Figure S10. Calibration curve showing a linear increase between the % SERS active area and the particle number concentration present in the standards. Error bars correspond to mean ± SD (n=2 replicates per condition with 3,600 spectra per replicate).

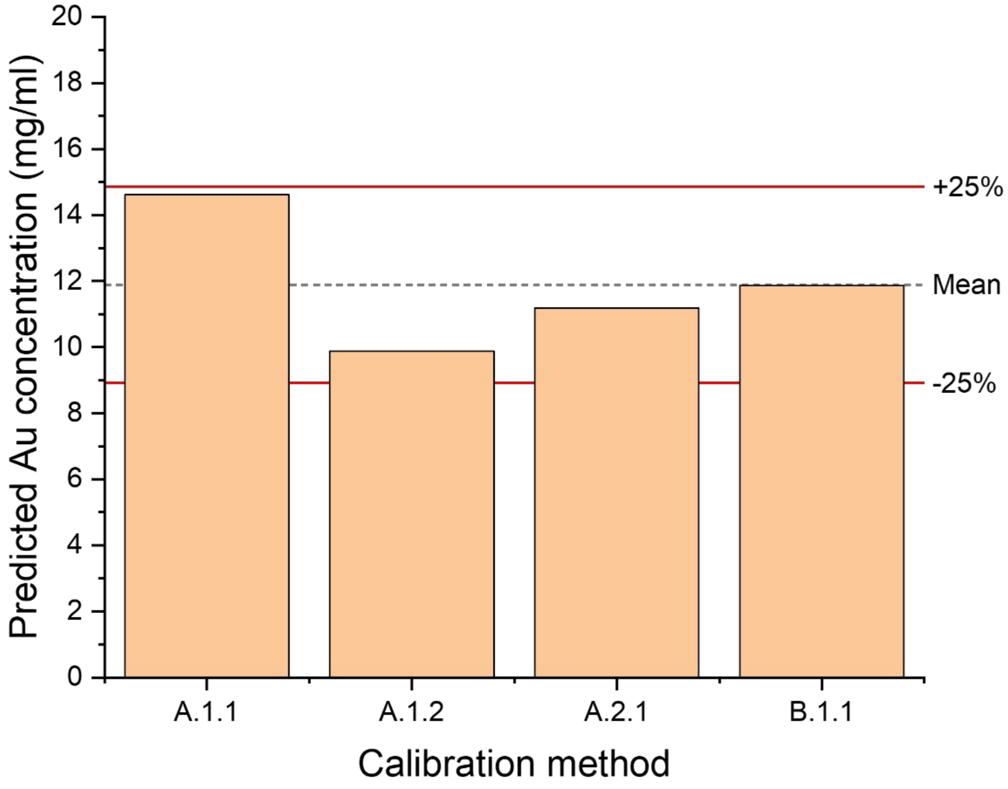

Figure S11. Comparison of the predicted Au concentrations per calibration method. The results show that all predictions are within the experimental error of 25%.
